# Heterogenetic parabiosis between healthy and dystrophic mice improve the histopathology in muscular dystrophy

**DOI:** 10.1038/s41598-020-64042-z

**Published:** 2020-04-27

**Authors:** Aiping Lu, Ping Guo, Liang Wang, Chieh Tseng, Matthieu Huard, Chris Allen, Ruth McCarrick-Walmsley, Kaitlyn E. Whitney, Johnny Huard

**Affiliations:** 1 Steadman Philippon Research Institute, Vail, CO 81657 USA; 2 Department of Neurology, The First Affiliated Hospital, Sun Yat-sen University, Guangzhou, GD 510080 China; 3 Department of Orthopaedic Surgery, The University of Texas Health Science Center at Houston, Houston, TX 77054 USA; 4 Department of Microbiology, Immunology and Pathology, Colorado State University, Fort Collins, CO 80523-1681 USA

**Keywords:** Muscle stem cells, Translational research

## Abstract

Duchenne muscular dystrophy (DMD) is a progressive muscle disease, characterized by mutations in the X-linked dystrophin, that has several therapeutic options but no curative treatment. Transplantation of muscle progenitor cells for treatment of DMD has been widely investigated; however, its application is hindered by limited cell survival due to the harmful dystrophic microenvironment. An alternative approach to utilize progenitor cells and circulatory factors and to improve the dystrophic muscle pathology and microenvironment is through parabiotic pairing, where mice are surgically sutured to create a joint circulatory system. Parabiotic mice were generated by surgically joining wild type (WT) mice expressing green fluorescent protein (GFP) with *mdx* mice. These mice developed a common circulation (approximately 50% green cells in the blood of *mdx* mice) 2-weeks after parabiotic pairing. We observed significantly improved dystrophic muscle pathology, including decreased inflammation, necrotic fibers and fibrosis in heterogenetic parabionts. Importantly, the GFP + cells isolated from the *mdx* mice (paired with GFP mice) underwent myogenic differentiation *in vitro* and expressed markers of mesenchymal stem cells and macrophages, which may potentially be involved in the improvement of dystrophic muscle pathology. These observations suggest that changing the dystrophic microenvironment can be a new approach to treat DMD.

## Introduction

Duchenne muscular dystrophy (DMD) is an X-linked progressive muscle wasting disease caused by a deficiency in dystrophin, leading to progressive myofiber necrosis, fibrosis and muscle weakness^1,2^. It is a fatal muscular dystrophy condition^3^ that affects 1 in 5000 adolescent males^4^. The progression of the disease leads to loss in ambulation (observed at 10–12 years of age), and premature death due to cardiac or respiratory failure (typically observed in early to mid-twenties)^5^. Despite years of considerable progress in understanding the molecular mechanism of the disease and advancements in therapeutic approaches^6^, there are currently no curative treatments for DMD. Nonetheless, several genetically engineered treatment modalities are currently being tested in pre-clinical and clinical trials as potential alternatives to conventional analgesic treatments (i.e., glucocorticoid steroid injections). Exon skipping is one of many genetic approaches that uses antisense oligonucleotides to restore the DMD reading frame by modulating the splicing process of dystrophyin^7–9^. However, current evidence does not support the therapeutic efficacy of exon-skipping drugs in DMD patients^8,10^. AAV carrying transgenes encoding for microdystrophin (µDYS) is a separate approach that has yielded up to 80% dystrophin-positive fibers and restored the Dystrophin Glycoprotein Complex (DGC) in treated *mdx* mouse muscles^10–12^. The results in the *mdx* mouse model have been conclusive and recent efforts have been focused on demonstrating the safety and efficacy of AAV vector coding for a functional µDYS in clinical trials^10^. CRISPR-Cas9-mediated genome editing has also been studied for the treatment of DMD because it can permanently replace the mutated dystrophin gene with the normal gene^13,14^; however, this modality faces several challenges before it can be safely translated and used clinically^15^. Moreover, genetically engineered treatment methods currently do not lead to full recovery of DMD patients. The use of progenitor stem cells is another alternative to genetically engineered and conventional treatment modalities to reset the microenvironment and recover dystrophin. Specifically, transplantation of muscle progenitor cells (MPCs) from healthy donors to treat DMD has been widely investigated^16–18^; however, the results are still not satisfactory. Researchers believe that its application is hindered by poor cell engraftment caused by the limited cell survival rate and immune-rejection^19–21^. In addition, the harmful microenvironment in dystrophic muscle is another challenge that hinders cell treatment and often results in poor transplantation outcomes. Little has been done to change the muscle microenvironment in DMD as a therapeutic approach to enhance stem cell therapy outcomes and rescuing the stem cell dysfunction observed in DMD.

For the last 20 years, the role of dystrophin and its restoration in mature muscle fibers has been the primary focus of DMD research^22–25^. Shifting the current paradigm, one study has recently shown that dystrophin is expressed in muscle satellite stem cells^26^. In fact, the lack of dystrophin expression in DMD has critical consequences for satellite cells including an inability to establish cell polarity, abrogation of asymmetric satellite stem cell divisions, and failure to enter the myogenic program^26^. This major finding corroborates with Chang *et al*.^27^ in that intrinsic satellite cell dysfunction exacerbates muscle wasting and ultimately impairs muscle fiber regeneration in dystrophic mice. Although it is still under debate whether satellite cell dysfunction in dystrophin-deficient mice is caused by intrinsic or extrinsic mechanisms^28–32^, it has been recently demonstrated that restoration of dystrophin using the CRISPR-Cas9 technique improves *mdx* MPC function *in vitro*.^33^ Additionally, MPCs isolated from DMD patients and dystrophic mice have been shown to be defective in their proliferation and differentiation capacities^29,34,35^. Therefore, it is critical to develop MPC-targeted therapeutic strategies to correct MPC dysfunction in DMD.

There are many ways to restore MPC function in DMD, including gene delivery of dystrophin, *in vivo* genome editing with CRISPR/Cas9, MPC transplantation. However, these therapeutic approaches are facing many limitations, such as low efficiency due to existence of inflammation and fibrosis in skeletal and cardiac muscle of DMD patients^15,36^. Even gene correction of dystrophin in the myocardium, which converts DMD to Becker Muscular Dystrophy (BMD, a milder form of the disease) at a later stage of the disease, cannot mitigate the inflammation and fibrosis in cardiac muscle of DMD patients^37,38^. In order to improve the therapeutic efficacy of MPC treatment, the dystrophic microenvironment may need to be modulated to be more conducive to cell therapies. Glucocorticoids, the current treatment standard for DMD, have been found to decrease the production and secretion of selected senescence-associated secretory components in human fibroblasts, including several pro-inflammatory cytokines IL-6, IL-8, GM-CSF and MCP-2^39^, indicating the beneficial effect of glucocorticoids for DMD patients likely acts through improving the dystrophic environment^40,41^. It has been shown that the levels of pro-inflammatory cytokines (IL-6, IL-1α, TNF-α) increased and the levels of anti-inflammatory cytokines (IL-4) decreased in the blood of *mdx* mice during the progression of the disease^42,43^. It also has been reported that exposure to factors present in the serum of young mice restores the regenerative capacity of aged MPCs^44^. Taken together, these results suggest that change in the dystrophic environment may delay the disease progression and enhance the outcome of therapeutic approaches for DMD.

Parabiotic pairing has been studied as a model for modifying the microenvironment and improving MPC function in the field of aging research^45^ but has not been used widely in the field of muscular diseases, such as DMD. In this study, we performed heterogenic parabiosis between healthy wild type (WT) and *mdx* mice. The *mdx* mice were joined with GFP transgenic mice to facilitate tracing the circulating cells from the GFP mice. We tested whether muscle histopathology can be improved by blood-borne factors and progenitor cells using a parabiotic system to enable a constant exchange of peripheral blood through microcirculation. Our results indicate a decrease in fibrosis, necrosis, and macrophage infiltration in the skeletal muscle of *mdx* mice after they were sutured with young WT mice for 8 weeks. In addition, we were able to isolate the GFP + cells from the *mdx* mice that were paired with WT-GFP mice for 8 weeks. Importantly, the GFP + cells have the potential to differentiate into a myogenic lineage *in vitro* and co-express markers of mesenchymal stem cells and macrophages, which may potentially be involved in modulating the dystrophic microenvironment. These observations suggest that altering the dystrophic microenvironment can be a new approach to alleviate muscle weakness in DMD models, despite the continued lack of dystrophin expression.

## Results

### Cross-circulation and redistribution of circulating cells from peripheral blood were established between two parabiotic mice

**Figure 1:**
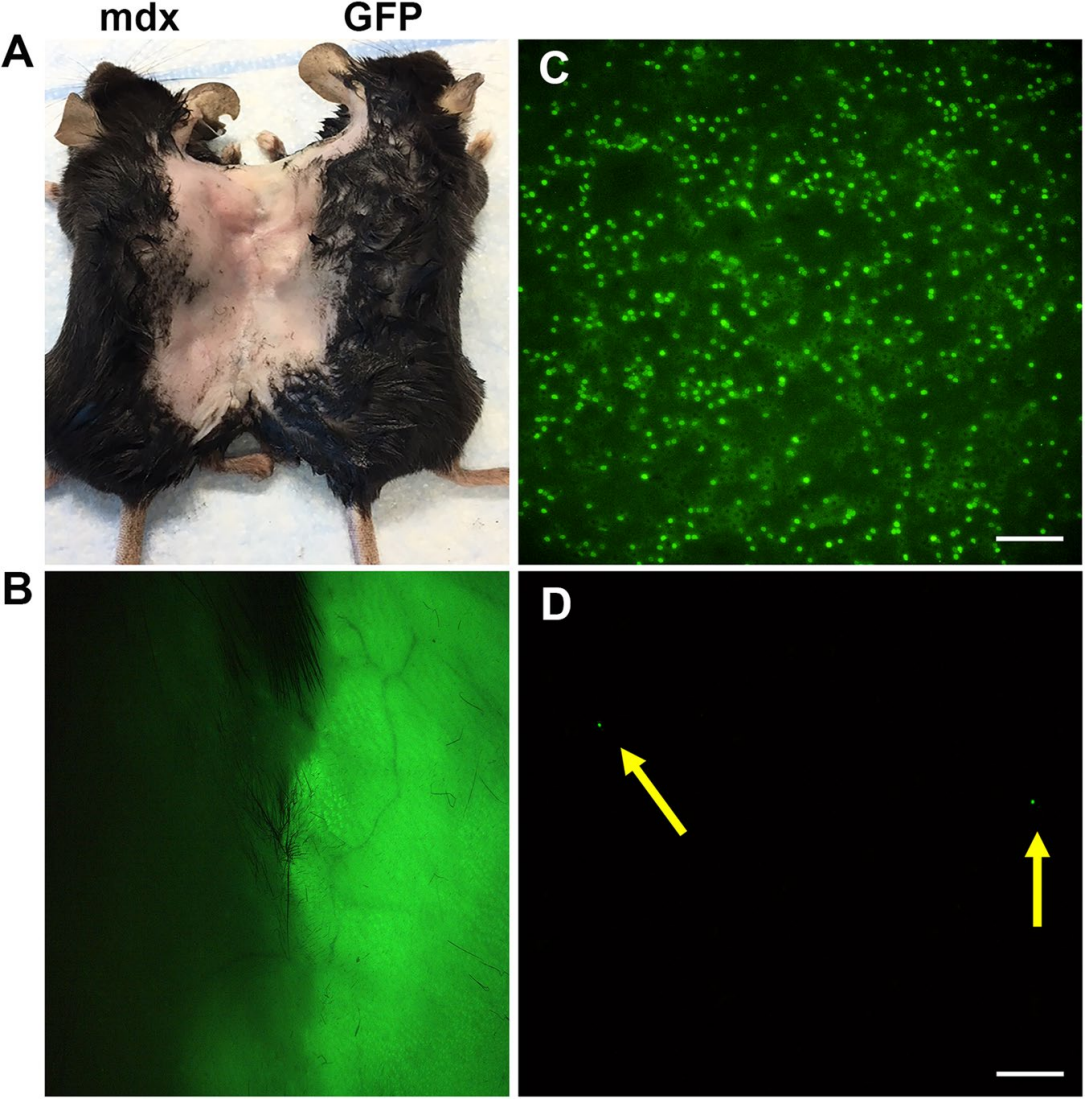
Confirmation of circulatory establishment between parabiotic pairings. (**A**) The representative image shows the heterogenetic pairing of *mdx* and WT-GFP mice. (**B**) Dissecting microscope image reveals peripheral blood in the skin of two mice. (**C**) Blood smear image from *mdx* mice that were paired with WT-GFP mice. (**D**) Blood smear image from *mdx* mice that were paired with *mdx* mice and injected green fluorescence beads via tail vein before sacrificed mice. The yellow arrows point to the green fluorescent beads. Scale bar = 50 µm (**C,D**).

To determine if common circulation can be established between pairs, we examined the peripheral blood of *mdx* mice that were parabiotically paired with GFP mice after the mice were sacrificed at 8 weeks post parabiosis surgery (Fig. 1A). Under the dissecting microscope, we were able to clearly observe the peripheral blood in the skin that connected two mice (Fig. 1B). Blood smears showed that 50% of blood cells of *mdx* mice were GFP positive, indicating the circulating system was shared between parabionts (Fig. 1C). Green fluorescent beads were injected into one of the isogenic *mdx* parabiotic pairs via the tail vein and then the peripheral blood of the collateral parabiotic partner was examined. Blood smears showed that the green fluorescence beads were also found in circulation of the non-injected *mdx* partner (Fig. 1D). Our results confirm the establishment of a shared circulatory system between parabiotic pairs, including heterogenic and isogenic pairs.

### Improved muscle histopathology in mdx mice parabiotically paired with GFP-WT mice

**Figure 2:**
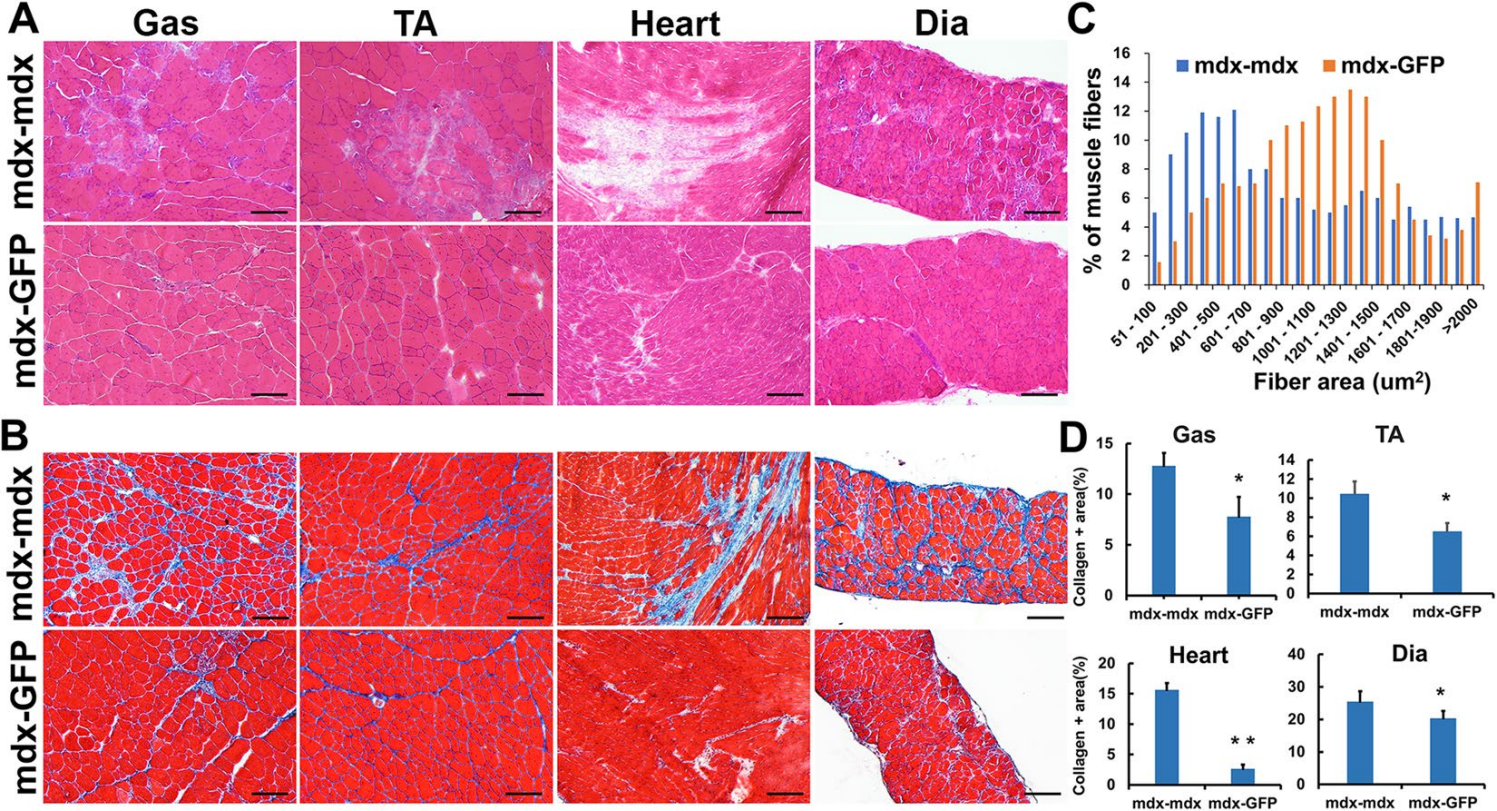
Improved muscle histology in *mdx* mice exposed to young WT peripheral circulation. (**A**) H&E staining and **(B)** trichrome staining, representative image shows the gastrocnemius, tibialis anterior, diaphragm muscle and cardiac tissue of *mdx* mice. **(C)** Quantification of the myofiber area between the parabiont *mdx* and control *mdx* muscle. The average number of myofibers of each size range calculated from >1,000 fibers analyzed per *mdx* muscle (n = 6). (**D**) Quantification of collagen positive area. Error bars indicate ‘mean + SD’, n = 6, *p < 0.05. Scale bar = 100 µm.

We performed parabiosis between WT-GFP mice and *mdx* mice (termed parabiont *mdx*). These heterogenic pairs were compared to the control isogenic pairs of two *mdx* mice (termed control *mdx*). The mice were euthanized at 8 weeks post-surgery and the gastrocnemius (Gas), tibialis anterior (TA), diaphragm (Dia) muscles and cardiac tissue were harvested. H&E staining showed the area of mononucleated cell infiltration and necrosis is smaller in the parabiont *mdx* muscles than the control *mdx* muscles (Fig. 2A), indicating that the skeletal muscles improved in their histopathological appearance. Trichrome staining showed there was also a significant reduction in muscle fibrosis in the parabiont *mdx* mice compared to control *mdx* mice (Fig. 2B,D, p < 0.05). This pathology improvement was observed in all the 3 skeletal muscles and cardiac tissues. We also noticed that the size of muscle fibers is consistently bigger in the parabiont *mdx* muscle than control *mdx* muscle (Fig. 2C**)**. This result indicates that heterogenetic parabiotic pairing improves dystrophic skeletal muscle histopathology.

### Heterogenetic parabiotic pairing attenuates inflammation in dystrophic skeletal muscle

**Figure 3:**
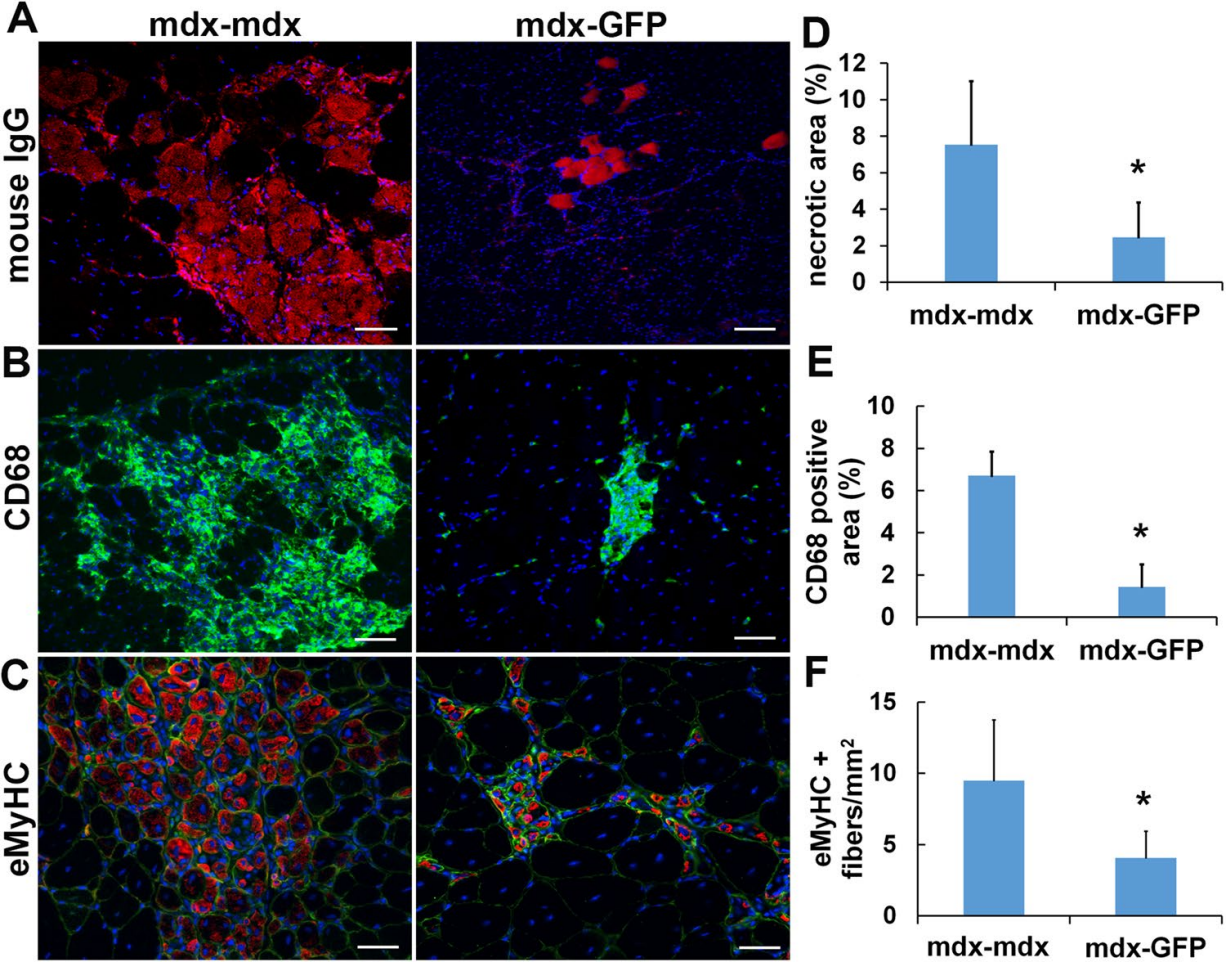
Heterogenetic parabiotic pairing decreased inflammation in dystrophic skeletal muscle. (**A**) Necrotic areas in the gastrocnemius muscles were identified by mouse IgG staining (red). The nuclei were stained with DAPI (blue). (**B**) Macrophage infiltration was identified by CD68 (green) staining. The nuclei were stained with DAPI (blue). (**C**) The new regenerated myofibers were identified by eMyHC (red) staining. The muscle fiber membrane was stained with laminin-a2 (green). (**D**) Quantification of necrotic fibers. (**E**) Quantification of CD68 positive area. (**F**) Quantification of eMyHC positive fibers. Error bars indicate ‘mean + SD’, n = 6, *p < 0.05. Scale bar = 50 µm (**A,B**). Scale bar = 25 µm (**C**).

To investigate the change in inflammation in the *mdx* muscle after parabiosis, immunofluorescent staining of gastrocnemius sections was performed. The results revealed a significant decline in mouse IgG+ necrotic fibers and CD68 + macrophage infiltration in the parabiont *mdx* muscle compared to the control *mdx* muscle (*P* < 0.05, Fig. 3A,B,D,E). As the embryonic isoform of myosin heavy chain (eMyHC) is present only in newly regenerated myofibers^34,46,47^, it is a valuable marker for muscle regeneration^47,48^. Therefore, we performed eMyHC immunofluorescence staining to test whether the reduction in inflammation by the heterogenetic parabiotic pairing has a role in dystrophic myofiber regeneration. As shown in Fig. 3C,F, our results indicate a significant reduction in eMyHC-positive myofibers in the muscles of *mdx* mice paired with WT-GFP mice after 8 weeks when compared with the muscles of control *mdx* mice. The muscle was co-stained with the laminin-a2 which is a protein in the basal lamina surrounding myofibers in order to visualize the muscle fibers. The ratio of eMyHC-positive fiber to total muscle fiber is 0.42 ± 0.12 for control *mdx* and 0.19 ± 0.06 for parabiont *mdx* muscles. As we know that eMyHC + fibers occur in areas of inflamed environment^34,49,50^, and these *mdx* mice are relatively young, a reduction in inflammation can also lead to a decrease in eMyHC-positive myofibers^51–53^. This explains the reduction of eMyHC-positive myofiber in the parabiont *mdx* mice.

### Heterogenetic parabiotic pairing improved dystrophic muscle environment and increased dystroglycan expression

**Figure 4:**
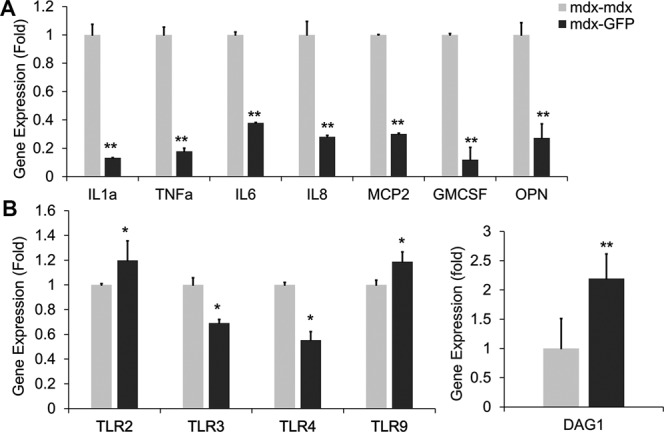
Heterogenetic parabiotic pairing decreased pro-inflammatory cytokines and increased dystroglycan expression. (**A**) Quantitative RT-PCR shows levels of gene expression for pro-inflammatory cytokines of the parabiont *mdx* and control *mdx* muscle. **(B)** Quantitative RT-PCR shows levels of gene expression for TLR signaling components of the parabiont *mdx* and control *mdx* muscle. **(C)** Quantitative RT-PCR shows levels of gene expression for DAG1 of the parabiont *mdx* and control *mdx* muscle. Error bars indicate ‘mean + SD’, n = 6, *p < 0.05. **p < 0.01.

Increased levels of pro-inflammatory cytokines exacerbate the dystrophic phenotype and down-regulation of pro-inflammatory cytokines correlates with an amelioration of the dystrophic phenotype in *mdx* mic^54–56^. To investigate if parabiotic pairing would change the dystrophic muscle environment by reducing the pro-inflammatory cytokine expression in muscle we next performed quantitative real-time PCR (qPCR). The results showed that pro-inflammatory cytokines IL-6, IL-8, GM-CSF, MCP-2, IL-1α and TNFα were significantly decreased in muscles of *mdx* mice after pairing with GFP mice (Fig. 4A, p < 0.01). These pro-inflammatory cytokines are involved in the progression of the disease in DMD and can be therapeutically targeted^54,55^. Osteopontin (OPN), a cytokine that promotes immune cell migration and survival^57^, is one of the most highly up-regulated genes in DMD patients and promotes fibrosis in dystrophic mouse muscle^57^. There is a significant reduction of OPN expression in parabiont *mdx* muscle compared to the control *mdx* muscle (Fig. 4A, p < 0.01). Toll-like receptors (TLR) signaling plays an important role in pathogenesis of dystrophic muscle and TLR antagonist significantly reduced skeletal muscle inflammation and increased muscle force of *mdx* mice^58–60^. We investigated the expression of TLR2, TLR3, TLR4 and TLR9 and the results indicated there is a significant reduction of TLR3 and TLR4 in the parabiont *mdx* muscle compared to the control *mdx* muscle (Fig. 4B, p < 0.05). However, the parabiosis did not show an effect on TLR2 and TLR9. TLR signaling plays a different role in skeletal muscle regeneration and pathogenesis of muscular dystrophy. TLR2 also is essential for skeletal muscle repair following acute injury^60^ and promotes angiogenesis^61^. Increased TLR2 and TLR9 expression may be due to a compensatory effect during homeostasis. The data from q-PCR clearly demonstrated that heterogenetic parabiotic pairing decreased pro-inflammatory cytokine expression which contributed to the dystrophic micro-environment and disease progression. This evidence demonstrates the beneficial effects of modifying the inflammatory milieu in dystrophic muscle^60^.

Although the deficiency of dystrophin is the primary cause for DMD, it has been observed that dystrophin-associated proteins are greatly reduced in skeletal muscle of DMD patients and *mdx* mice^62–64^. In order to evaluate whether the parabiotic pairing affects DGC proteins, we performed qPCR for DAG1, a gene that encodes dystroglycan, which is one of the dystrophin-associated glycoproteins. Interestingly, the qPCR result demonstrated increased DAG1 expression at the mRNA level in parabiont *mdx* muscle compared to control *mdx* muscle (Fig. 4C), indicating that changes to the environment will increase the levels of DGC-related proteins.

### Identification of GFP + cells isolated from the mdx muscle after pairing with WT mice

**Figure 5:**
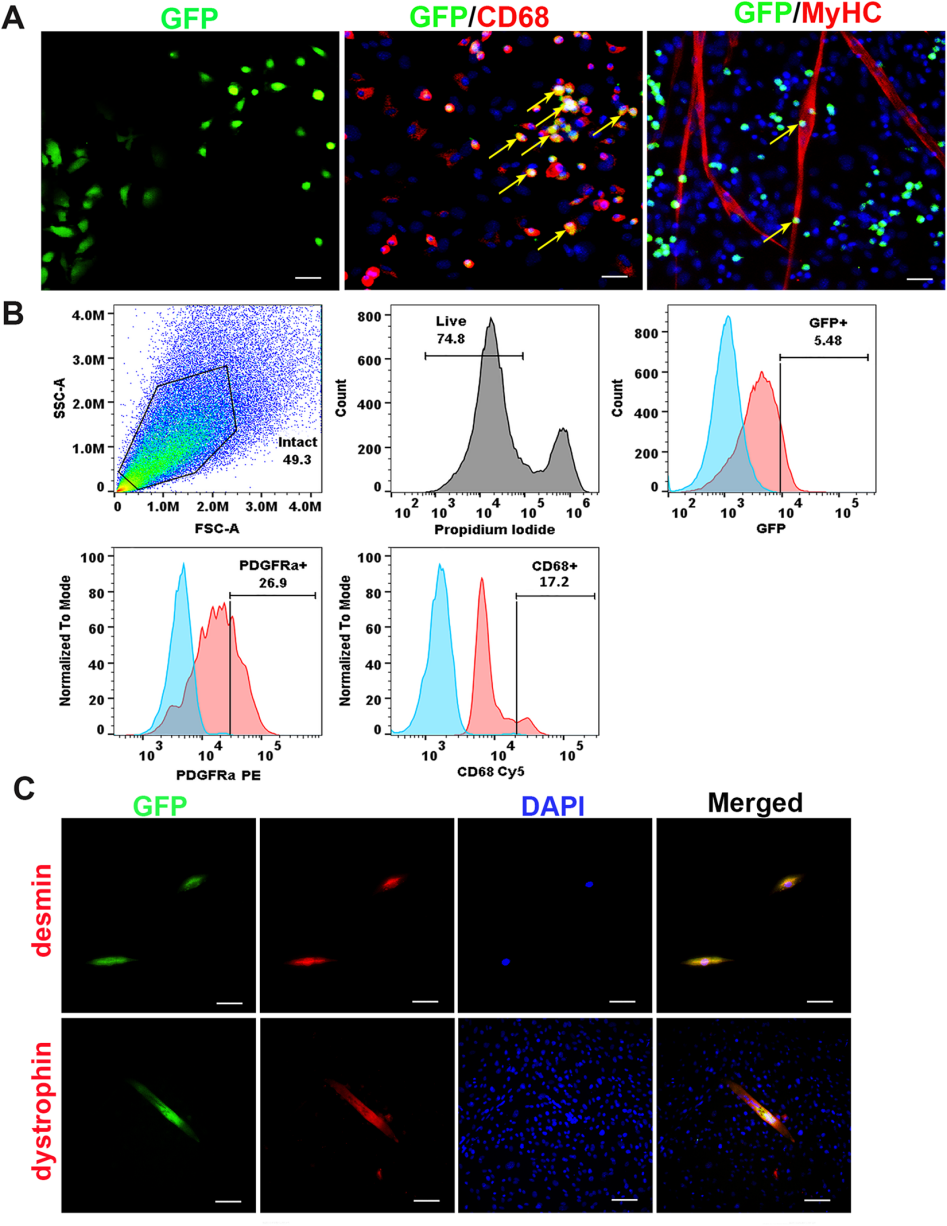
Identification of GFP + cells in the *mdx* muscle *in vitro*. (**A**) Representative image of GFP positive cells isolated from *mdx* mice paired with GFP mice (GFP, green). Yellow arrows indicate GFP + cells that are colocalized with CD68 (red), and not colocalized with MyHC (red). The nuclei were stained with DAPI (blue). (**B**) Flow cytometric analyses of the GFP + circulating cells isolated from the muscle of parabiont *mdx* mic*e*. (**C**) Representative images show the desmin staining (top, red) and the colocalization with GFP (green). The myotubes were stained with dystrophin (red, bottom) after 3 days differentiation. Representative images show the colocalization with GFP (green). The nuclei were stained with DAPI (blue). In panel A, scale bar = 50 µm for GFP, scale bar = 25 µm for CD68 and MyHC. In panel B, scale bar = 25 µm for desmin, scale bar = 50 µm for dystrophin).

Parabiosis has been used to characterize blood or bone marrow–borne circulating cells in response to tissue injuries^65–68^. To investigate whether GFP + cells from the WT partner would migrate to the *mdx* muscle after stabilization of cross-circulation, we isolated the muscle cells from parabiont *mdx* mice using the preplate technique^69^. We observed GFP + cells present not only in the fast adhering cell populations, but also in the slow adhering cell populations. The GFP + cells display different size and morphology (Fig. 5A**, GFP**). GFP + cells were characterized by immune staining for surface markers. The results showed that 10 ± 4.2% of GFP + cells were positive for CD68, a macrophage marker (Fig. 5A**, GFP/CD68, yellow arrows**). Very few GFP + cells (<1%) were found in MyHC+ myotubes after cultivation in proliferation medium, but these do not co-express MyHC (Fig. 5A**, GFP/MyHC, yellow arrows**). Flow cytometry analysis indicated that 5.48 ± 1.4% of GFP + cells were found in the fast adhering cell populations after culturing *in vitro*. 17.2 ± 4.2% GFP + cells co-expressed CD68 and 26.9 ± 4.6% of GFP + cells are PDGFRα + , a marker of mesenchymal stem cells (Fig. 5B). Importantly, when muscle cells from the *mdx* partner of heterogenetic pairings were cultured in myogenic differentiation medium for 3 days we found that GFP + cells (1.5 ± 0.3%) co-expressing desmin, a myogenic progenitor cell marker (Fig. 5C**, top**), and some GFP + cells (1.7 ± 0.5%) in the fast adhering cell populations were able to differentiate into myotubes. Dystrophin staining confirmed those myotubes are dystrophin + /GFP + (Fig. 5C**, bottom**), further confirming that these GFP + cells have a capacity to differentiate into myogenic lineage *in vitro*. This result suggests that the circulating GFP + cells from the WT-GFP partner of heterogenetic pairs migrate to the *mdx* muscle. Additionally, the GFP+ cells  co-express PDGFRα and a macrophage marker and demonstrate myogenic differentiation potential. The circulating GFP + cells may play a role in reducing inflammation and improving the muscle histopathology in *mdx* mice through different mechanisms and may also be involved in muscle regeneration.

### Characterization of GFP + cells in the mdx muscle after pairing with WT mice

**Figure 6:**
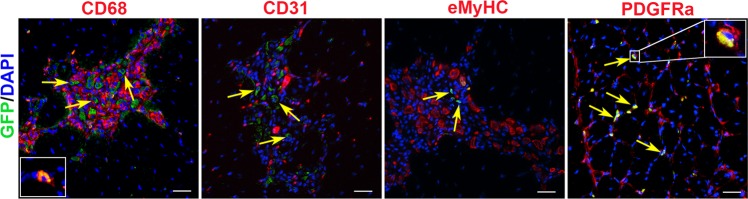
Identification of GFP + cells in the *mdx* muscle *in vivo*. Representative images show the muscle sections from the parabiont *mdx* mice were co-stained GFP (green) with CD68, CD31, eMyHC and PDGFRα (red). The cell co-expressing CD68 and GFP is shown in the left bottom of CD68 staining. The nuclei were stained with DAPI (blue). The yellow arrows indicate the GFP + cells. A representative enlarged image (right top corner) in PDGFRα staining panel shows the cell co-expressing PDGFRα and GFP. Scale bar = 25 µm.

Parabiosis allows a shared circulatory system to be established in two surgically conjoined animals and is suitable to assess the contribution of circulating cells to tissue repair^68^. We next sought to characterize the GFP + cells *in vivo* in order to determine if GFP + cells directly participate in muscle repair. We detected 15 ± 3.5 GFP + cells per muscle section in the skeletal muscle of parabiont *mdx* mice (Fig. 6). The immunostaining result showed that only a few GFP + cells (1 ± 0.3%) were positive for CD68 (Fig. 6**, CD68**, shown in the left bottom of CD68 staining). The majority of GFP + cells were not colocalized with CD68 **(**Fig. 6**, CD68)**. GFP + cells did not co-express CD31 (Fig. 6**, CD31**), a marker of pericytes and endothelial cells. More importantly, we did not find the GFP + cells that co-expressed eMyHC (Fig. 6**, eMyHC**) in the parabiont *mdx* muscle, suggesting that the GFP + cells do not directly participate in muscle regeneration. Interestingly, we identified 80 ± 5.2% of GFP + cells that colocalized with PDGFRα in the parabiont *mdx* muscle (Fig. 6**, PDGFRα**). Our results suggested that GFP + cells are not directly involved in muscle regeneration but may release factors which are necessary for the process.

## Discussion

Although various therapeutic approaches for DMD have been investigated, there is currently no cure for this disease. For the last 20 years, the restoration of dystrophin has been extensively investigated through a variety of cell and gene therapy approaches^70^. Recent evidence has shown that dystrophin plays a role in stem cell function^26^ and, more importantly, in the onset of the disease correlated with stem cell depletion/exhaustion^34^. These results taken together suggest that DMD is also a stem cell disease^26,33^.

Parabiotic pairing has been used for many years to modulate the microenvironment in aged muscle that consequently improves the function of aged MPC^44^. In order to determine if disease-related loss of MPC function is primarily driven by cell autonomous and/or non-autonomous mechanisms and to further develop new approaches to improve DMD patients’ quality of life without restoring dystrophin, we have performed parabiotic pairing between *mdx* and WT mice in an attempt to modulate the microenvironment. These experiments were motivated by the question of whether blood-borne factors/progenitor cells from young healthy WT mice can change the dystrophic microenvironment and improve the histopathology of *mdx* mice. We found that pro-inflammatory cytokines IL-6, IL-8, GM-CSF, MCP-2, IL-1α and TNFα significantly decreased and there was also a significant reduction in OPN expression in parabiont *mdx* muscle compared to the control *mdx* muscle. It has been previously reported that these cytokines and OPN promote progression of DMD^54,55,57,71,72^. GM-CSF plays a major role in macrophage proliferation and survival in the muscle and reduction of GM-CSF expression can result in decreased macrophage infiltration in the *mdx* muscle. Our results indicate that heterogenetic parabiotic pairing of healthy mice with dystrophic mice alleviates DMD muscle histopathology predominately through reducing inflammation and fibrosis. These parabiotic results provide some evidence to suggest that the dysfunction in MPCs is driven by the dystrophic microenvironment and might be attributable to changes in circulating factors. In addition, the histological improvement may be related to a beneficial effect imparted on the dystrophic MPCs by the circulating factors from the young WT mice.

It has been shown that dystrophin-associated proteins are greatly reduced in skeletal muscle from *mdx* mice^63^. We investigated whether parabiotic pairing will affect the levels of various DGC-related proteins. Interestingly, we found significant increases in the level of dystroglycan mRNA expression in the parabiont *mdx* muscle compared to the control group. It has been reported that the level of utrophin, a homolog of dystrophin, is increased in skeletal muscle of *mdx* mice, which may account for reduced pathogenesis in *mdx* mice compared with DMD patients^73,74^. We also performed immune staining for dystrophin and utrophin on the parabiont *mdx* muscles and control *mdx* muscles but found no significant differences (data not shown).

These observations demonstrate that changes in the dystrophic microenvironment can provide a new target for improving muscle weakness in DMD. However, the identity of circulating factors and stem/progenitor cells contributing to this beneficial effect  is still unknown. Although some previous studies have demonstrated that circulating cells can contribute to tissue repair^65–68,75^ after the shared circulation was established, other researchers believe there is insufficient evidence of blood cell contribution to tissue regeneration^45,76^. In this study, we observed that the circulating GFP + cells migrated from WT-GFP mice to the muscle of *mdx* mice after subjecting them to parabiosis for 8 weeks. We did not detect circulating GFP + cells giving rise to an observable quantity of muscle fibers *in vivo*. However, those WT-GFP + cells are capable of differentiating into a myogenic lineage *in vitro*. Interestingly, we found some GFP + cells that co-express the mesenchymal stem cell marker PDGFRα and macrophage marker CD68 in the muscle of parabiont *mdx*. We suspect that they are inflammatory cells, mesenchymal and hematopoietic stem cells (HSCs). The regenerative potential of mesenchymal stem cells was confirmed not only by their ability to differentiate into diverse tissues but also by their immunomodulatory and anti-inflammatory properties through secretion of a variety of growth factors and anti-inflammatory cytokines^77^. It has also been demonstrated in previous studies that bone marrow cells can reconstitute muscle, but the identity of the cells and the mechanism of differentiation remains unknown. In two studies which performed lineage tracing of HSCs transdifferentiating to muscle tissue, the first demonstrated that single HSCs can generate skeletal muscle through myeloid intermediates^78^, and the second proved that single HSCs can give rise to both blood and muscle cells^79^. In our study, we have not yet fully explained the exact role of the WT-GFP cells which colonize *mdx* muscle in heterogenetic pairings. Additional *in vivo* and *in vitro* studies are needed to further determine if these cells improve the DMD muscle pathophysiology by paracrine or by direct mechanisms.

We also noticed that the muscles of GFP mice that were paired with *mdx* mice for 8 weeks did not exhibit any observable abnormalities (data not shown). This effect has not been reported in the aging research field using the heterogenetic pairing model. Dr. Irina M. Conboy’s group reported that the inhibitory effects of blood from old mice are more pronounced than the beneficial effects of blood from young mice, and that peripheral tissue injury compounds the negative effects^80^. This phenomenon can be explained by the fact that the environment and circulating factors are different between mouse models which are naturally aged and those with genetic disease. Additional research is necessary to identity these circulating factors and progenitor cells. In this study, we did not perform GFP-GFP isogenic pair controls since the GFP mice that were paired with *mdx* mice did not exhibit observable muscle abnormalities. Future muscle injury studies in GFP mice are necessary to investigate how the *mdx* environment affects WT muscle regeneration.

**In conclusion**, our findings provide evidence that circulating factors or cells following parabiotic pairing could improve the muscle histopathology of *mdx* mice. Although the identity of the circulating factors and progenitor cells requires further examination. We believe these results will provide insights that will advance the development of therapies that can rescue stem cell defects in muscular dystrophy and may delay disease progression in DMD. However, parabiosis can only serve as a proof of concept and cannot be used clinically. Exchange transfusion is a potential life-saving procedure that is employed to counteract the effects of serious jaundice or changes in the blood due to diseases, such as sickle cell anemia^81^. The procedure involves slowly removing the person’s blood and replacing it with fresh donor blood or plasma. This method has been widely used in clinical practice for many decades. In addition, heterochronic blood exchange in small animals^80^ have been shown to be less invasive and enables better-controlled studies with more immediate translation to therapies for humans. In future studies, we will perform a blood exchange between young WT and dystrophic mice, with and without transplanting stem cells from healthy donors, to determine if blood exchange will demonstrate beneficial effects to improve the function of muscle stem cells in dystrophic mice. We hypothesize that changing the muscle microenvironment in DMD patients by exchange transfusion would not only improve the function of muscle stem cells, but also alleviate the muscle weakness in DMD.

## Materials and Methods

### Animals

The C57BL/6-Tg (UBC-GFP) 30Scha/J (termed GFP-WT) mice are transgenic mice which express enhanced Green Fluorescent Protein (GFP) under the control of the human ubiquitin C promoter. C57BL/10ScSn-Dmdmdx/J (mdx) mice have a C57BL/10ScSn genetic background with a dystrophin mutation at exon 23 on the X chromosome. Both GFP and *mdx* mice were purchased from Jackson Laboratory. All animal studies and related experimental protocols were approved by the University of Texas’s Animal Care and Use Committee. The methods were performed in accordance with the approved guidelines and regulations.

### Parabiosis surgery

Three-month-old gender-matched GFP and *mdx* mice were prepared for surgery. The parabiotic surgery was conducted following the procedure as previously described^45^. Briefly, a matching incision was made from the elbow joint of the forelimb to the knee joint of the hindlimb of each animal. Each animal pair was sutured with ligaments and skin ligated between partners using 4–0 nylon suture. Animals were given buprenorphine after surgery. Mice were sacrificed at 8 weeks post parabiosis surgery. Two age-matched *mdx* mice were used as the control isogenic pairs, while WT to *mdx* pairs were the experimental heterogenetic parabionts. WT-GFP mice are wild type mice that ubiquitously express a green fluorescent protein reporter gene in order to track the circulating cells. For *mdx-mdx* control pairs, green fluorescent beads were injected into one *mdx* mouse by tail vein before the mice were sacrificed for confirmation that conjoined animals shared a network of blood vessels. The blood smears were made immediately after the mice were sacrificed. A total of 6 GFP-*mdx* pairs and 6 *mdx-mdx* control pairs (4 female and 2 male) were analyzed in this study. The person preforming the analysis was blinded.

### Histology

Eight weeks after parabiosis the mice were sacrificed, and the skeletal muscles, including gastrocnemius, tibialis anterior, diaphragm and cardiac tissue were harvested, and flash frozen in liquid nitrogen-cooled 2-methylbutane^82^. H&E and trichrome staining were performed on 10 µm cryosections from gastrocnemius muscles (GM) according to the manufacturer’s instructions. All stained sections were visualized on a Nikon Eclipse E800 fluorescence microscope^34^. Six pictures were randomly selected per slide and the collagen positive areas were measured and quantified (positive area per total area of muscle section (%)) using ImageJ software^34^. To determine the fiber size, the muscle fiber cross-sectional area was measured for every fiber in each section using ImageJ software. A total of 6 muscles from each group were analyzed.

### Isolation of muscle cells from mdx paired with WT-GFP mice

Muscle cells were isolated from *mdx* mice that were paired with WT-GFP mice for 8 weeks using a modified preplate technique^69,83^. Briefly, skeletal muscle tissue was minced and processed through a series of enzymatic dissociations: 0.2% of collagenase type XI (Sigma-Aldrich, C7657) for 1 hour, 2.4 units/ml of dispase (Invitrogen, 17105–04) for 45 minutes, and 0.1% of trypsin-EDTA (Invitrogen, 15400–054) for 30 minutes at 37 °C. After enzymatic dissociation, the muscle cells were centrifuged and resuspended in proliferation medium (Dulbecco’s modified Eagle’s medium (DMEM, Invitrogen, 11995–073) supplemented with 10% fetal bovine serum (FBS, Invitrogen, 10437–028), 10% horse serum (HS, Invitrogen, 26050–088), 0.5% chicken embryo extract (CEE, Accurate Chemical Co, CE650T-10), and 1% penicillin-streptomycin (Invitrogen, 15140–122). The cells were then plated on collagen type I (Sigma-Aldrich, C9791) coated flasks. Different populations of muscle-derived cells were isolated based on their adhesion characteristics^84^.

### Myogenic differentiation assay and fast myosin heavy chain staining

The muscle cells were plated on 24 well plates (30,000 cells/well) in DMEM supplemented with 2% FBS to promote myogenic differentiation (myotube formation). Three days after plating, immunocytochemical staining for fast myosin heavy chain (MyHCf) was performed. After rinsing two times with PBS, cells were fixed for 5 minutes in cold methanol (−20 °C), blocked with 10% donkey serum (Jackson ImmunoResearch, 017–000–121) for 1 hour, and then incubated with a mouse anti-MyHCf (Sigma-Aldrich, M4276, 1:250) antibody for 2 hours at RT. The primary antibody was detected with an Alexa 594-conjugated anti-mouse IgG antibody (Invitrogen, A21203, 1:500) for 30 minutes. The nuclei were revealed by 4, 6-diamidino-2- phenylindole (DAPI, D9542, 100 ng/ml, Sigma-Aldrich) staining. The percentage of differentiated myotubes was quantified as the number of nuclei in MyHCf positive myotubes relative to the total number of nuclei.

### RNA isolation and qRT-PCR assay

Total RNA from parabiont *mdx* muscles and control *mdx* muscles was isolated using TRizol Reagent (Invitrogen) and reverse transcribed using the iScript reverse transcription supermix cDNA synthesis kit (Bio-Rad) according to the manufacturer’s protocol. Real-time PCR was carried out using the Applied Biosystems™ SYBR™ Green Assay kit (Thermo Fisher) and an Applied Biosystems StepOnePlus Real-Time PCR thermocycler (Applied Biosystems). Primers were designed using PRIMER-Blast (NCBI) and their sequence has been described in Table 1.

**Table 1:** **Primer Sequences**.

Name	Sequence
IL-1a	Forward: 5′-ccgtgttgctgaaggagttg-3′, Reverse: 5′-AGGTGCACCCGACTTTGTTCTT-3′
IL-6	Forward: 5′-agtggctaaggaccaagacc-3′, Reverse: 5′-tctgaccacagtgaggaatg-3′
IL-8	Forward: 5′-CTCCATGGGTGAAGGCTACT-3′, Reverse: 5′-TGTTCTCAGGTCTCCCAAATGA-3′
GMCSF	Forward: 5′-aagaagccctgaacctcctg-3′, Reverse: 5′-ctggtagtagctggctgtca-3′
OPN	Forward: 5′-TCCCTCGATGTCATCCCTGTTG-3′, Reverse: 5′-GGCACTCTCCTGGCTCTCTTTG-3′
TLR2	Forward: 5′-CACCATTTCCACGGACTGTGGTACCTG-3′, Reverse: 5′-cagcttaaagggcgggtcagagtt-3′
TLR3	Forward: 5′-GACTGGGTCTGGGAACATTTCTCC-3′, Reverse: 5′-GCTTgctgaactgcgtgatgtacc-3′
TLR4	Forward: 5′-ATCTACTCGAGTCAGAATGAGGACTGG-3′, Reverse: 5′-GGctgctcagaaactgccatgt-3′
TLR9	Forward: 5′-ctgggacgtctggtactgttttca-3′, Reverse: 5′-CAGCTCGTTATACACCCAGTCGGC-3′
MCP2	Forward: 5′-CAGTGCTTCTTTGCCTGCTG-3′, Reverse: 5′-ggggcactggatattgttgatt-3′
DAG1	Forward: 5′-cttccttagcaactggtggc-3′, Reverse: 5′-tcggagagaactgagtgcat-3′
GAPDH	Forward: 5′-TGGCAAAGTGGAGATTGTTGCC-3′, Reverse: 5′- AAGATGGTGATGGGCTTCCCG-3′

### Immunohistochemical analyses in vitro and ***in vivo***

Skeletal muscle cryosections were fixed in 5% formalin or 4% paraformaldehyde for 10 minutes and pre-incubated in 10% donkey serum (017–000–121, Jackson ImmunoResearch) in PBS for 1 hour at RT. The cryosections were incubated for 3 hours at RT with primary antibodies for CD68 (Abcam, ab53444,1:300) and GFP (Abcam, ab290,1:500), CD31 (BD Pharmingen, 557355, 1:300), PDGFRα (R&D, AF1062, 1:200), laminin-a2 (Sigma, L0663,1:200), washed in PBS, and then incubated for 30 minutes at RT with secondary antibodies: 488-conjugated donkey anti-rat IgG (Invitrogen, A21208,1:500), 594-conjugated donkey anti-rat IgG (1:500; Invitrogen, A21209), 594-conjugated donkey anti-mouse IgG (Invitrogen, R37115, 1:500) and Alexa 488-conjugated donkey anti-rabbit IgG (Invitrogen, A21206, 1:500). A Mouse On Mouse kit (Vector, BMK-2202) was used for mouse IgG and eMyHC staining according to the manufacturer’s protocol. The eMyHC primary antibody (1:50) from Developmental Studies Hybridoma Bank (F1.652 C) was used. Cy3-streptavidin (1:500, Sigma-Aldrich, GEPA43001) was added to act as the tertiary antibody. The nuclei were revealed by 4, 6-diamidino-2- phenylindole (DAPI, D9542, 100 ng/ml, Sigma-Aldrich) staining. The cells isolated from *mdx* muscle were seeded into a 24-well plate (3×10^4^ cells/well) and cultured in proliferation medium and, 24 hours later, the cells were fixed in 5% formalin for 5 minutes and pre-incubated in 10% donkey serum (Jackson ImmunoResearch,017–000–121) in PBS for 1 hour at RT. The cells were then incubated for 3 hours at RT with primary antibodies for dystrophin (Abcam, ab15277, 1:300) and Desmin (Invitrogen, PA5–16705, 1:300), washed in PBS, and then incubated for 30 minutes at RT with secondary antibodies Alexa 594-conjugated donkey anti-rabbit IgG (Invitrogen, A21207, 1:500) and Alexa 594-conjugated donkey anti-rat IgG (Invitrogen, A21209,1:500). The nuclei were stained with DAPI. A negative control for any antibody staining was used in which the first antibody is omitted from the immunostaining. All stained sections were visualized on a Nikon Eclipse E800 fluorescence microscope. We also determined the exposure time, based on the lack of staining in the negative control, when the pictures were taken. Six pictures were randomly selected per slide, and the GFP + cells from parabiont *mdx* mice and eMyHC+ fibers were manually counted based on 20x and 10x images, respectively. The eMyHC+ fibers were quantified using ImageJ software. The mouse IgG+ necrotic fibers and CD68 + macrophage infiltration areas were measured and quantified (positive area per total area of muscle section (%)) using ImageJ software.

### Flow cytometric analyses

Early preplate cells isolated from the parabiont *mdx* mice were cultured in proliferation medium for 3–5 passages.

The cells were then stained with fluorochrome-conjugated antibodies against PDGFRα (PE-conjugated, Biolegend, 135905) and CD68 (PerCP/Cyanine5.5 conjugated, Biolegend,137009) on ice for 30 minutes. Spectral cytometry was performed using Cytek® Aurora (Cytek Biosciences) at Colorado State University Flow Cytometry Core Facility. FlowJo was used for data analysis.

### Statistical analysis

All results are given as the mean ± standard deviation (SD). Means from isogenic pairs and experimental heterogenetic parabionts mice were compared using Student’s *t-*test. Differences were considered statistically significant when the *P* value was <0.05.

## Data Availability

All data generated or analyzed during this study are included in this published article.
